# Pre‐existing CD95‐based Temra immunity in patients with recurrent/metastatic nasopharyngeal carcinoma predicts response and hyperprogression to dual PD‐L1 and TGFβ inhibition

**DOI:** 10.1002/ctm2.70535

**Published:** 2025-11-20

**Authors:** Ngar Woon Kam, Jeffrey Yan Ho Lau, Cho Yiu Lau, Tai Chung Lam, Kenneth Sik Kwan Chan, Wei Dai, Victor Lee Ho Fun, Chi Leung Chiang, Dora Lai Wan Kwong

**Affiliations:** ^1^ LKS Faculty of Medicine The University of Hong Kong Hong Kong China; ^2^ School of Pharmacy, Faculty of Medicine The Chinese University of Hong Kong Hong Kong China; ^3^ Department of Clinical Oncology, Centre of Cancer Medicine, School of Clinical Medicine, LKS Faculty of Medicine The University of Hong Kong Hong Kong China; ^4^ Clinical Oncology Center The University of Hong Kong‐Shenzhen Hospital Shenzhen China

## Abstract

None.

## Graphical abstract

• Lower baseline CD95⁺ Temra levels were associated with treatment response and longer survival.

• Higher CD57⁺ Temra frequencies predicted improved overall survival, independent of response.

• Post‐treatment shifts in CD95/CD57 expression correlated with survival outcomes.

• TGFβlow/CD95⁺ Temra high profile identified non‐responders with hyperprogression risk.

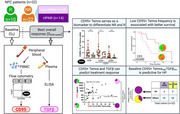

1

Dear Editor,

Limited success with immunotherapy alone has prompted trials combination therapies, which were reported to enhance tumour infiltration and immune memory.^1^ Identifying key effector memory cells and the optimal immune environment pre‐treatment is crucial. We previously reported a phase II study (NCT04396886) using bintrafusp alfa, a dual inhibitor of TGFβ and PD‐L1, in 38 resistant recurrent/metastatic nasopharyngeal carcinoma (R/M NPC) patients. Overall response rate was 23.7% response rate, but with an exceptionally high hyperprogression (HP) rate of 21.1%.^2^ This companion study examined T‐cell memory traits at baseline (D_0_) and at the time of best overall response (D_BOR_), exploring their link to activation status of circulating terminally differentiated effector memory T cells re‐expressing CD45RA (Temra), plasma TGFβ, treatment responses, and HP.

This study analyzed 64 PBMC samples from 32 R/M NPC patients, assessed after at least 60 days of treatment, with a BOR of 31% (Figure 1A; Table S1 and Supporting Information Methods). Patient characteristics are summarized in Table S2. Memory T‐cell subsets were defined based on CCR7 and CD45RA expression^3^ (Table S3, Figure S1A; Figure 1B), enabling classification into naïve, central memory, effector memory, and Temra cells. Flow cytometric analysis revealed that non‐responders showed no significant change in CD45+ cells (Figure S1B), but exhibited a decrease in CD3^+^ (total) and CD8^+^ T‐cell subsets (CD3^+^/CD8^+^; Figure 1C). On the contrary, responders demonstrated a significant increase in total CD45^+^ leukocytes (Figure S1B) and CD4+ T‐cell subsets (CD3^+^CD4^+^) following treatment (Figure 1D). To identify immune correlates of treatment response, we performed sparse partial least squares discriminant analysis (sPLS‐DA) on post‐treatment memory T‐cell subsets. This analysis identified CD8+ Temra cells (CD3^+^/CD8^+^/CD45RA^+^/CCR7^−^) as the most discriminative feature, with the largest negative loading separating responders from non‐responders (Figure 1E; Table S4), suggesting their potential role in treatment outcomes. Given the relevance of CD95 and CD57 to effector T‐cell homeostasis^4^ and immunosenescence,^5^ respectively, we next examined their expression within Temra subsets to further characterize functional phenotypes associated with response (Figure 2A).

**FIGURE 1:**
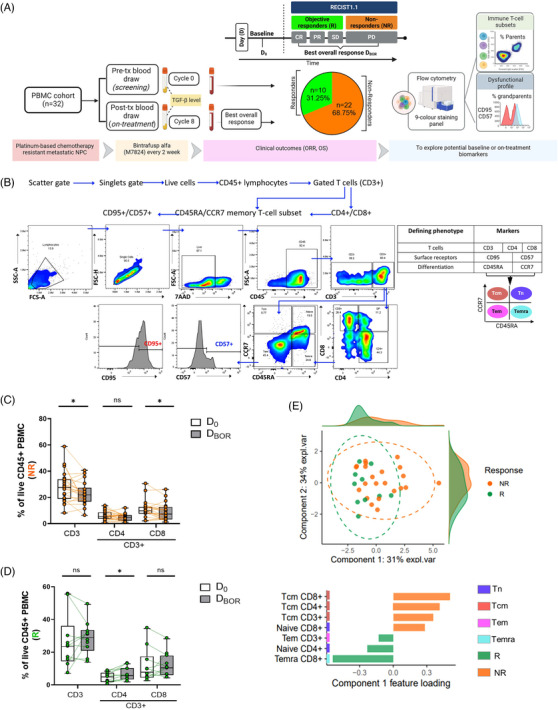
Bintrafusp alfa treatment response tends to correlate with higher levels of circulating Temra cells. (A) Flowchart of 32 patients with metastatic or recurrent NPC treated with TGFβ/PD‐L1 co‐blockade. (B) Schematic of CD3⁺, CD4⁺, and CD8⁺ T‐cell subsets analyzed by flow cytometry at D_0_ and D_BOR_ using fluorescence‐minus‐one gating. Subsets (naïve, Tcm, Tem, Temra) defined by CCR7 and CD45RA expression. (C, D) Boxplots showing frequencies of total CD3^+^, CD3^+^/CD4^+^ and CD3^+^/CD8^+^ of gated live CD45+ lymphocytes of NR (C; orange, *n* = 22) and R patients (D; green, *n* = 10) at D_0_ and D_BOR_. (E) sPLS‐DA performed on post‐treatment immune cell subsets in R (green) vs NR (orange). Top: Component score plot (Comp 1: 31% explained variance, Comp 2: 34% explained variance) with 95% confidence ellipses and marginal density distributions for each component. Bottom: component 1 feature loadings; Temra CD8+ cells showed the largest negative loading, enriched in R. *p < .05. Boxplots, centerline = medians, boxes: 25th–75th percentiles. Statistical comparisons differences between BOR groups and time points were determined using unpaired and paired *T*‐test, respectively. ORR, overall response rate; OS, overall survival; R, responders; NR, non‐responders; HP, hyperprogressors; CR, complete response; PR = partial response; SD, stable disease; PD, progressive disease; D_BOR_, day of best overall response; D_0_, baseline; Tn, naïve; Tcm, T‐cell central memory; Tem, T‐cell effector memory; Temra, T‐cell effector memory re‐expressing CD45RA.

**FIGURE 2:**
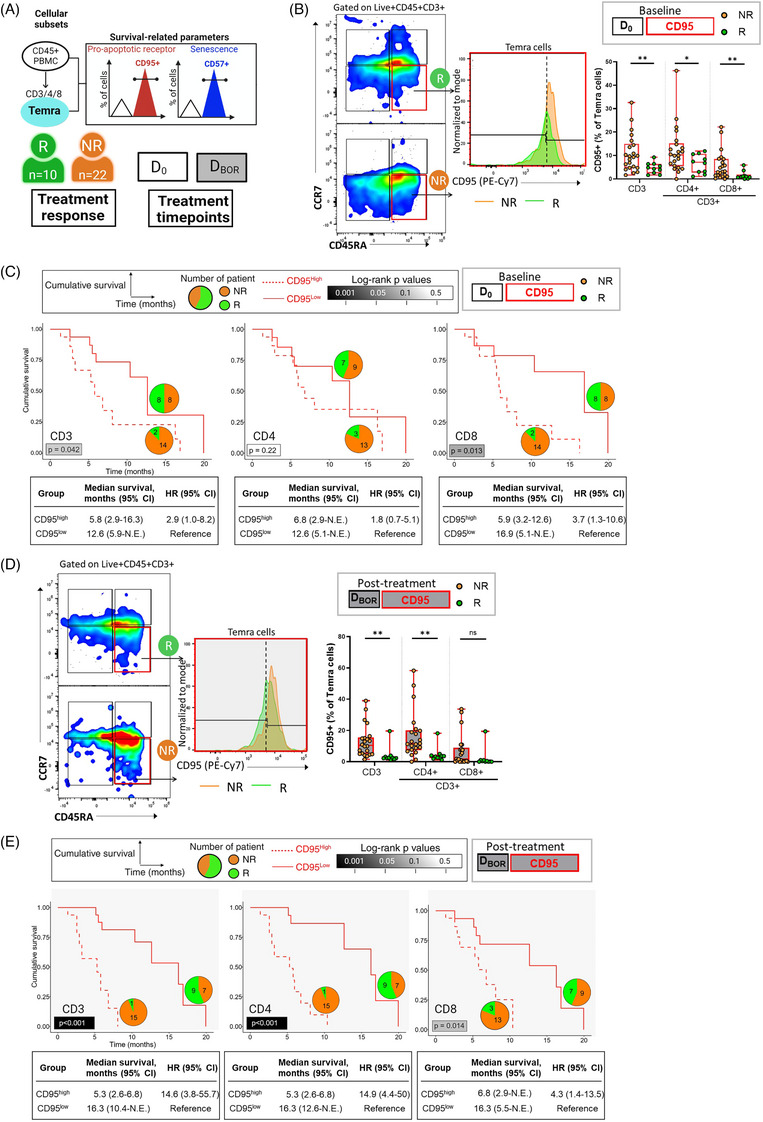
Lower frequencies of CD95‐expressing Temra T cells prior to and after therapy are associated with patient response to TGFβ and PD‐L1 blockade. (A) Study design and gating strategy. Live CD45+ lymphocytes were gated for CD3^+^, CD4^+^, and CD8^+^ subsets, followed by memory classification using CD45RA/CCR7 and phenotyping with CD95/CD57. (B) Representative CD95 staining in total CD3+ Temra cells from responders (*n* = 10) vs. nonresponders (*n* = 22; left), and CD95 frequencies in Temra cells before treatment (right). (C) Kaplan–Meier plot showing overall survival based on CD95 expression (above/below median) in CD3/4/8^+^ Temra cell at D_0_. (D) Representative on‐treatment CD95 frequencies in CD3+ Temra cells from responder and non‐responder patients. (E) Survival comparison by CD95 expression at D_BOR_: median OS (months) for high D_BOR_ vs. low CD95 Temra cells—CD3^+^: 5.3 vs. 16.3 (*p* < .001); CD4^+^ cells: 5.3 vs. 16.3 (*p* < .001); CD8^+^: 6.8 vs. 16.3 (*p* = .014). *p < .05, **p < .01. Boxplots show medians and interquartile ranges. Unpaired t‐tests were used for group comparisons; log‐rank tests for survival analysis. Color coding: responders (green), non‐responders (orange), D_0_ (white), D_BOR_ (shaded), CD95 (red), CD57 (blue). CI, confidence interval; NE, not estimable.

At baseline (D_0_), responders exhibited significantly lower frequencies of CD95^+^ Temra cells compared with non‐responders (Figure 2B; total (CD3^+^CD45RA^+^/CCR7^−^), median% 4.61% ± 2.38% vs. 9.53% ± 7.90%, *p* = .0022; CD4^+^ (CD3^+^/CD4^+^/CD45RA^+^/CCR7^−^), median%7.10% ± 4.01% vs. 10.2% ± 9.91%, *p* = .0266; CD8+(CD3^+^/CD8^+^/CD45RA^+^/CCR7^−^), median% 0.985% ± 1.82% vs. 3.21% ± 6.04%, *p* = .0073). Patients were then categorized into high and low CD95 expression groups on Temra cells, using the median value as the cutoff. Those with lower CD95 levels had significantly longer overall survival (OS): median OS of 5.8 vs. 12.6 months for total Temra (*p* = .042), and 5.9 vs. 16.9 months for CD8^+^ Temra (*p* = .013) (Figure 2C). The majority of CD95‐high total and CD8^+^ Temra cells were found in non‐responders (87.5%), while responders accounted for only 12.5%. CD95‐high CD4⁺ Temra cells were less predictive but still more prevalent in non‐responders (81.25%). We also compared EBV DNA levels between CD95^+^ Temra‐high and Temra‐low groups. No statistically significant differences were observed (Table S5; Figure S1C), although patients with lower pre‐treatment EBV DNA levels tended to have better survival outcomes (Figure S1D), as previously reported.^2^ Interestingly, responders also tended to have higher CD57^+^ Temra levels (*p* = .071; Figure S2A). Patients with higher baseline CD57^+^ Temra had significantly longer survival than the low group (median OS: 12.6 vs. 3.8 months, *p* = .035; Figure S2B). While most CD57‐low patients were non‐responders (81.3% and 75% in CD4^+^ and total/CD8^+^, respectively), baseline CD57^+^ levels appeared to be more strongly associated with survival than treatment response.

Post‐therapy (D_BOR_), responders showed significant reductions in CD95 expression within total and CD4+ Temra cells (*p* < .005), with a trend towards decreased CD8^+^ Temra cells (*p* = .1169; Figure 2D; Table S6). In contrast, CD57 expression was elevated in responders at D_BOR_ (Figure S2C; Table S7) and correlated with improved OS (Figure S2D). Consistently, high D_BOR_ CD95 in total, including both CD4^+^ and CD8^+^ Temra was associated with worse OS (Figure 2E), while high D_BOR_ CD57 Temra in total and CD4^+^ Temra cells predicted better OS (Figure S2B). The majority of non‐responders exhibited high D_BOR_ CD95 (93.8% and 81.3% in total/CD4^+^ and CD8^+^ Temra cells, respectively) and low D_BOR_ CD57 expression (81.3% and 75% in total/CD4^+^ and CD8^+^ Temra cells, respectively).

Given the inverse correlation between CD95 and CD57 expressing profiles in our cohort (Figure S3), we speculate that a CD95lowCD57high Temra pattern may have clinical relevance. Notably, patients exhibiting this pattern, particularly within CD4^+^ Temra cells, demonstrated improved survival, while non‐responders largely lacked this pattern (D_0_: 22.7% and 27.3% in total/CD8+ and CD4+ Temra cells, respectively; D_BOR_: 18.2%, 13.6% and 22.7% in total, CD4^+^ and CD8^+^ Temra cells, respectively; Figure S4A,B). To further investigate the clinical significance of CD95 and CD57 expression on Temra cells, we examined their association with HP, which was observed in 14 of 22 non‐responders (63.6%, referred to as HPNR; Figure 3A). Among non‐responders without HP (non‐HPNR), CD95^+^ Temra cells showed minimal phenotypic changes between D_0_ and D_BOR_, with a trend towards increased CD57 in CD4^+^ Temra cells (Figure 3B). Contrary, HPNR patients exhibited increased CD95+ Temra in total, particularly within the CD4+ subset, and unchanged or decreased CD57 expression at D_BOR_ (Figure 3C). We next assessed pre‐treatment plasma TGFβ levels to explore their potential link to response. Although non‐HPNR had higher TGFβ levels at D_0_ than HPNR, levels in HPNR were comparable to those in responders (Figure 3D). At D_week8_, responders had significantly higher TGFβ levels (mean ± SD, 175.2 ± 137.0 ng/mL) than non‐HPNR (5.034 ± 18.84 ng/mL), while most HPNR patients showed undetectable TGFβ. Notably, 20% responders also had undetectable TGFβ, indicating post‐treatment TGFβ depletion alone does not fully explain HP events.

**FIGURE 3:**
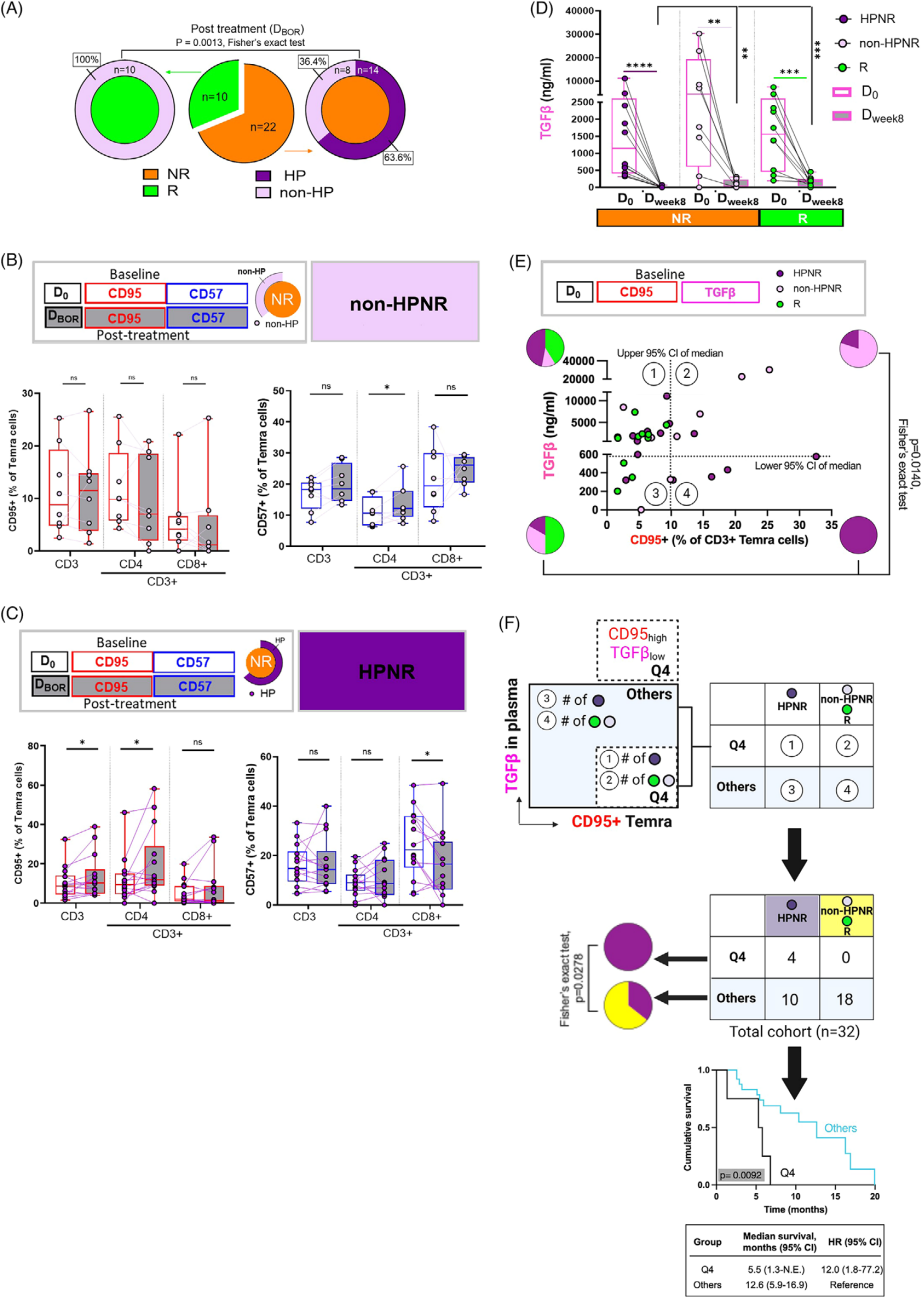
Frequencies of CD95‐expressing Temra T cells and plasma TGFβ is predictive to treatment outcome. (A) Pie charts summarizing patient distribution. Middle: overall frequencies of responders (*n* = 10) and non‐responders (*n* = 22). Left: inner circle shows all responders; outer circle shows proportion without HP (non‐HP; *n* = 10). Right: inner circle shows all non‐responders; outer circle shows proportions with (non‐HPNR; *n* = 14) and without HP (HPNR; *n* = 8). (**B, C**) Frequencies of CD95 (left) and CD57 (right) on gated live CD45+ CD3/4/8+ Temra cells on baseline (D_0_) and on‐treatment (D_BOR_) in non‐responders without HP (non‐HPNR; B) and with HP (HPNR; C) (D) Plasma TGFβ levels before and after treatment across groups (median [range ng/mL]): non‐HPNR group (median 4370 ng/mL [range 0–30 260 ng/mL]); HPNR group (median 1151 ng/mL [320.8–11 090 ng/mL]); responders (median 1567 ng/mL [201.4–7406 mg/mL]). (E) Quadrant‐based stratification using 95% CI thresholds: lower bound of TGFβ (578 ng/mL) and upper bound of CD95⁺ Temra frequency (9.89%) at D_0_. Dashed lines indicate CI boundaries (plasma TGFβ and CD95⁺ Temra cell frequencies at D_0_ (CD95‐expressing Temra: 95% CI 4.74–9.890; TGFβ: 95%CI 578–2518); pie charts show response frequencies per quadrant. (F) Validation of Q4 enrichment using CI‐based cutoffs across the full cohort. Pie charts show patient distribution in Q4 versus the rest of the cohort. Q4 was significantly enriched in hyperprogressors (*p* = .0278; OR = 15.86), and associated with shorter overall survival (*p* = .0092; HR = 12.0). Boxplots display medians and interquartile ranges. Statistical comparisons used unpaired and paired *t*‐tests; survival analysis by log‐rank test. *p < .05, **p < .01, ***p < .001, ns = not significant.

To further investigate biomarker associations, we stratified patients using scatter plots based on the 95% confidence interval (CI) of median for D_0_ plasma TGFβ levels and CD95^+^ Temra frequencies. This approach defined four biomarker quadrants, with Quadrant 4 (Q4) TGFβlow/ CD95^+^ Temra high profiles. Fisher‐exact test revealed that patients in Q4 were predominantly HPNR, while responders were mainly found in Quadrant (Q1), characterized by TGFβlow CD95^+^ Temra low profiles (Figure 3E). The distribution of response types across quadrants differed significantly (*p* = .0140), although this stratification did not specifically identify HP among non‐responders. To validate the association, we compared the frequency of Q4 profiles against the remaining cohort. Approximately 13% of HPNR patients fell into the pre‐defined Q4 category (Figure 3F), and this enrichment was statistically significant (Fisher's exact test, *p* = .0278; odds ratio: 15.86, 95% CI:.78–324.4), supporting the link between the Q4 biomarker profile (TGFβlow/ CD95^+^ Temra high) and HP. Kaplan–Meier curves revealed a statistically significant difference in OS between Q4 and other patients (Figure 3F; *p* = .0092). Q4 patients had a median survival of 5.5 months (95% CI: 1.3–N.E.), compared with 12.6 months (95% CI: 5.9–16.9) in others. The hazard ratio for Q4 was 12.0 (95% CI: 1.8–77.2), indicating a markedly increased risk of poor outcome.

Although our findings are primarily descriptive, they suggest mechanistic links between Temra phenotypes and the dual PD‐L1 and TGFβ blockade. The enrichment of CD95⁺ Temra cells in non‐responders and hyperprogressors may reflect a population of terminally differentiated T cells prone to activation‐induced cell death (AICD),^4^ potentially exacerbated by PD‐L1 inhibition in the absence of sufficient TGFβ‐mediated immune regulation. Beyond its apoptotic role, CD95 can also activate alternative pathways such as NF‐kB and PI3K/Akt, contributing to immune dysfunction and tumour progression.^6^ This dual functionality highlights the importance of contextual interpretation when evaluating CD95 expression in relation to treatment resistance and HP. Although higher CD95 expression was associated with non‐responders, the lack of data on the metalloprotease generating soluble CD95L limits full characterization of the CD95/CD95L axis. In contrast, CD57+ Temra cells—enriched in responders—are known to express high levels of cytolytic molecules such as granzyme/perforin expression.^7^ Given their reported stability and slower turnover,^8^ CD57^+^ Temra cells may represent “long‐lived effectors” capable of sustained anti‐tumour activity, with potential as biomarkers for durable immune responses^9^ and improved survival.^10^ The observed inverse relationship between CD95 and CD57 expression suggests a functional dichotomy within the Temra compartment, potentially shaped by PD‐L1 and TGFβ signalling. We hypothesize that dual inhibition may selectively favour expansion or survival of CD57⁺ Temra cells while failing to suppress CD95‐mediated exhaustion or apoptosis in non‐responders. Further mechanistic studies are warranted to dissect how PD‐L1 and TGFβ pathways modulate Temra cell fate and function, and whether CD95/CD57 profiling can inform therapeutic strategies that enhance effector persistence while minimizing HP. Together, these data suggest that profiling CD95 and CD57 expression may offer a valuable strategy for early risk stratification and personalized treatment in NPC.

Currently, no biomarkers are available for pre‐treatment selection of NPC patients for immunotherapy, and responses have been observed regardless of PD‐L1 or EBV DNA levels.^11^, ^12^ In our cohort, a CD95low/CD57high profile was associated with better prognosis, though the limited sample size and use of bintrafusp alfa alone may affect generalizability. Validation in larger cohorts and with other checkpoint inhibitors is needed to clarify whether the observed effects reflect PD‐L1 inhibition, TGFβ inhibition, or their interaction. Plasma TGFβ levels may also underestimate biological activity due to poor detection of exosomal TGFβ, which remains active in R/M cancer patients.^13^ While prior studies have linked Temra cells to immunotherapy response in head and neck cancers,^14^ our study provides a more in‐depth characterization by profiling CD95 and CD57 expression within Temra subsets. We found that CD95^+^ Temra cells were associated with poor outcomes, whereas CD57^+^CD8^+^ Temra cells correlate with improved survival, highlighting functional heterogeneity within the Temra compartment (Figure 4). These findings deepen our understanding of T‐cell functionality in cancer immunotherapy and underscore the need for robust biomarkers in the context of dual checkpoint blockade.

**FIGURE 4:**
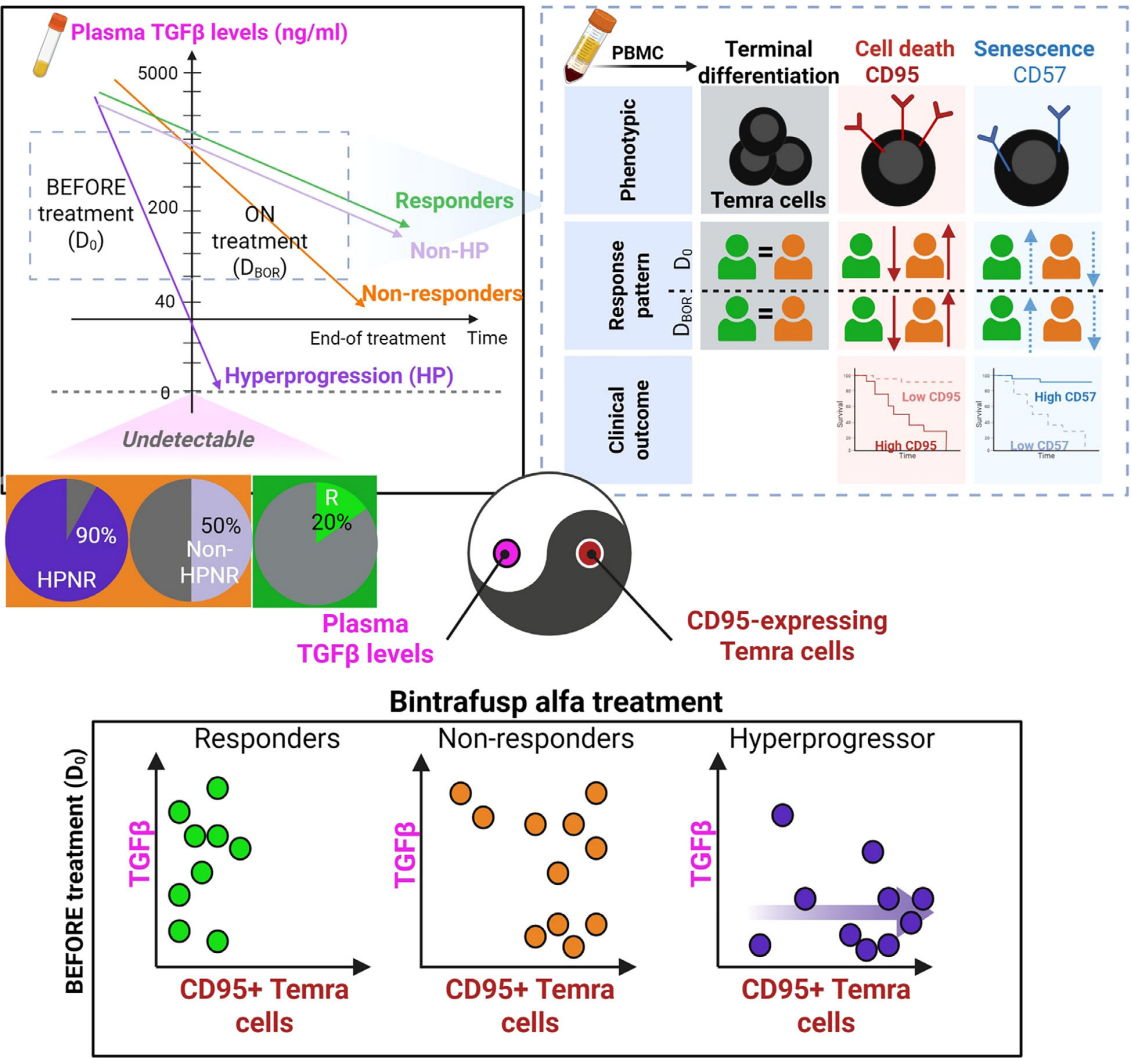
Conceptual schematic illustrating a proposed hypothesis for potential mechanisms related to treatment response and hyperprogression (HP) during Bintrafusp alfa therapy in R/M NPC patients, Top left panel: Illustrative summary of plasma TGFβ patterns at D_week8_ in responders (green) and non‐responders (orange), further sub‐grouped into non‐responders without HP (light purple) and with HP (dark purple). The pie chart represents the percentage of patients in each respective color category with undetectable TGFβ levels at week 8 of treatment. Top right panel: Summary of the predictive value of CD95 and CD57 expression on Temra cells for before (D_0_) and post‐treatment at D_BOR_. Bottom panel: Proposed relationship at (D_0_) between HP risk and combined markers (low plasma and high CD95 expression on Temra). D_BOR_ = day of best overall response.

PD‐L1 expression remains the most clinically relevant biomarker for guiding checkpoint inhibitor therapy in head and neck cancers, including NPC. However, its predictive value is complicated by technical variability, specimen type, and intratumoral heterogeneity. Studies have shown that PD‐L1 expression can vary depending on biopsies versus resection specimens, primary versus metastatic site, and timing relative to conventional therapies such as platinum‐based chemotherapy.^15^, ^16^, ^17^ These findings underscore the limitations of PD‐L1 as a static biomarker and highlight the need for complementary immune profiling strategies. Our study suggests that CD95 and CD57 expression within Temra subsets may offer a more dynamic and functionally relevant alternative, particularly in the context of dual PD‐L1 and TGFβ blockade. This approach may help overcome the challenges associated with PD‐L1 heterogeneity and better stratify patients for personalized immunotherapy. Altogether, our findings support the integration of Temra subset profiling into future immunotherapy strategies and contribute to a broader immunological framework that favours combination approaches targeting both immune checkpoints and immunosuppressive cytokines, advancing precision immuno‐oncology.

## AUTHOR CONTRIBUTIONS

Ngar Woon Kam: designed and conducted experiments and responsible for conceptualization. Jeffrey Yan Ho Lau: analyzed experiments, data interpretation, and conceptualization. Cho Yiu Lau and Wei Dai: visualization. Tai Chung Lam, Kenneth Sik Kwan Chan, and Chi Leung Chiang: collected data and ran the clinical trial. Victor Lee Ho Fun and Dora Lai Wan Kwong: funding acquisition. Dora Lai Wan Kwong: supervised the study. Ngar Woon Kam: wrote the original draft. Ngar Woon Kam, Jeffrey Yan Ho Lau, Cho Yiu Lau, Wei Dai, and Dora Lai Wan Kwong: reviewed and revised the manuscript. The order of co‐first authors was assigned by the contribution to this study. The order of co‐corresponding authors was determined by the fact that Dora Lai Wan Kwong served as the principal investigator of the HMRF funding grant, Chi Leung Chiang served as the principal investigator of the clinical trial and Victor Lee Ho Fun served as the team leader of the Health@InnoHK Program.

## CONFLICT OF INTEREST STATEMENT

The authors declare no conflict of interest.

## ETHICS STATEMENT

This study was approved by The University of Hong Kong/Hospital Authority Hong Kong West Cluster Institutional Review Board (IRB number: UW 19–675). The patients/participants provided their written informed consent to participate in this study.

## Supporting information



Supporting Information

Supporting Information

Supporting Information

Supporting Information

Supporting Information

Supporting Information

Supporting Information
