# Supplementary figures and images for: Pre‐existing CD95‐based Temra immunity in patients with recurrent/metastatic nasopharyngeal carcinoma predicts response and hyperprogression to dual PD‐L1 and TGFβ inhibition
Source: Clin Transl Med. 2025 Nov 20;15(11):e70535. doi: 10.1002/ctm2.70535 (PMC12632158; doi:10.1002/ctm2.70535)

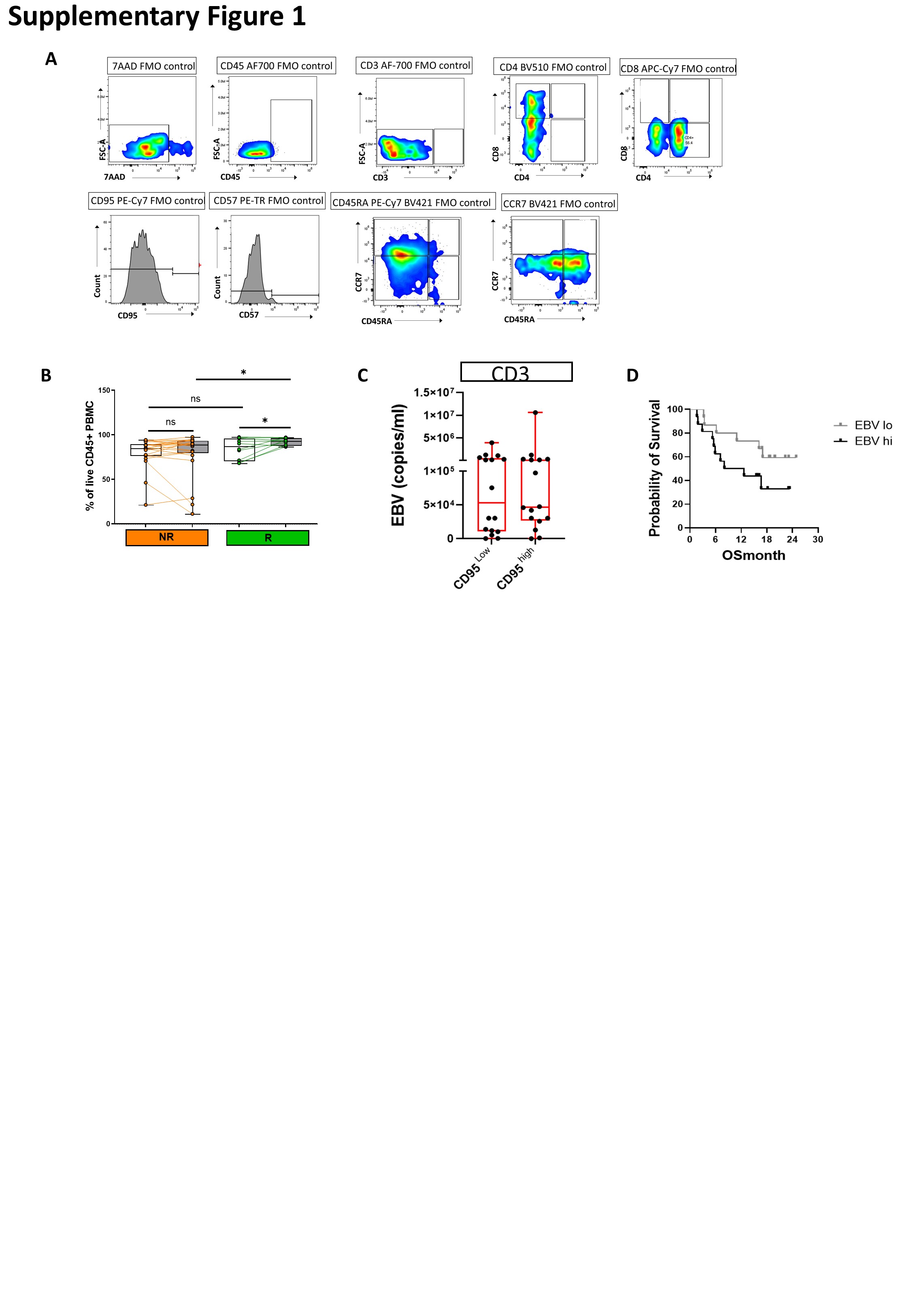

Supplement: Supplementary file 1 — Supporting Information [file CTM2-15-e70535-s003.jpg]

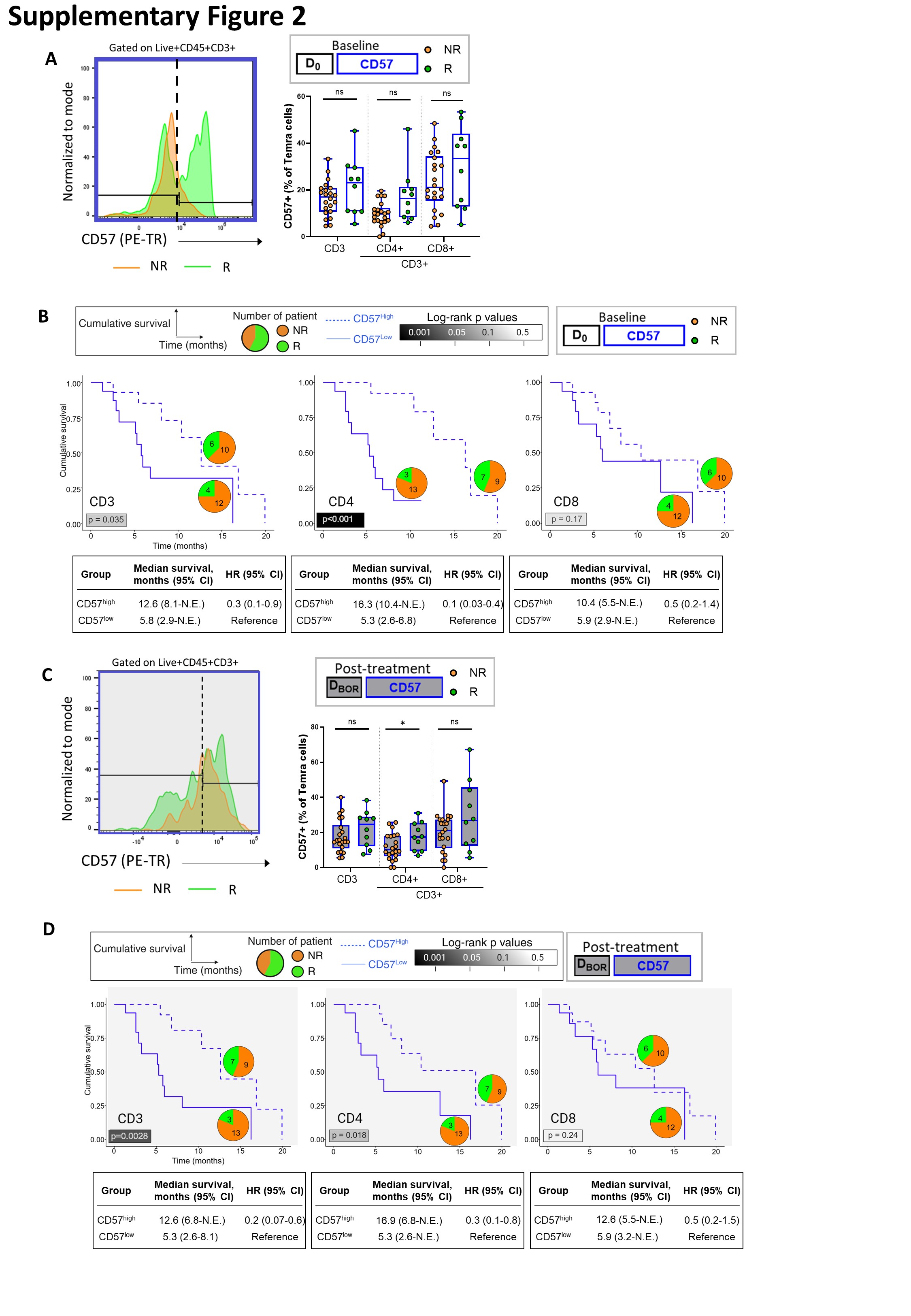

Supplement: Supplementary file 2 — Supporting Information [file CTM2-15-e70535-s001.jpg]

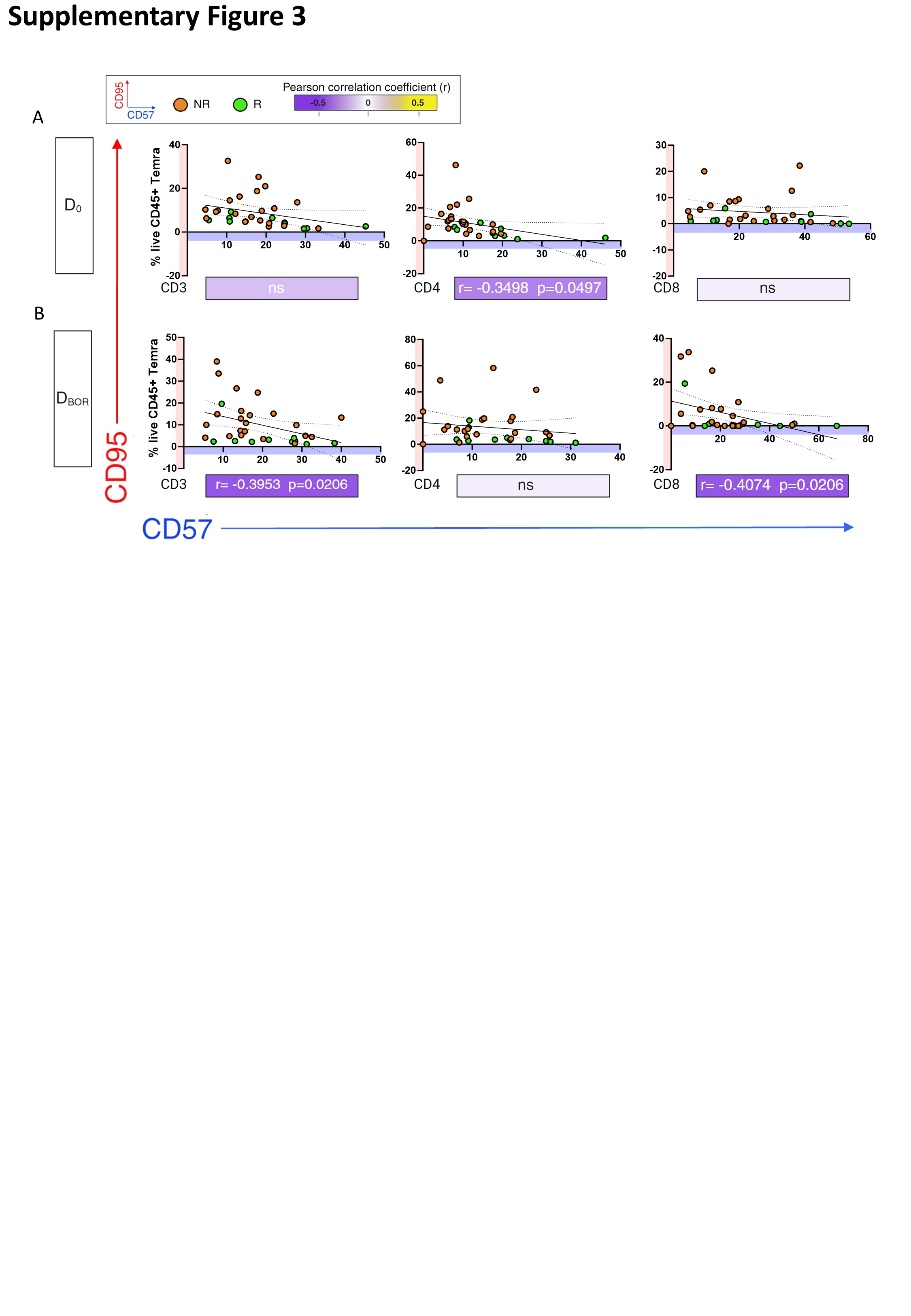

Supplement: Supplementary file 3 — Supporting Information [file CTM2-15-e70535-s006.jpg]

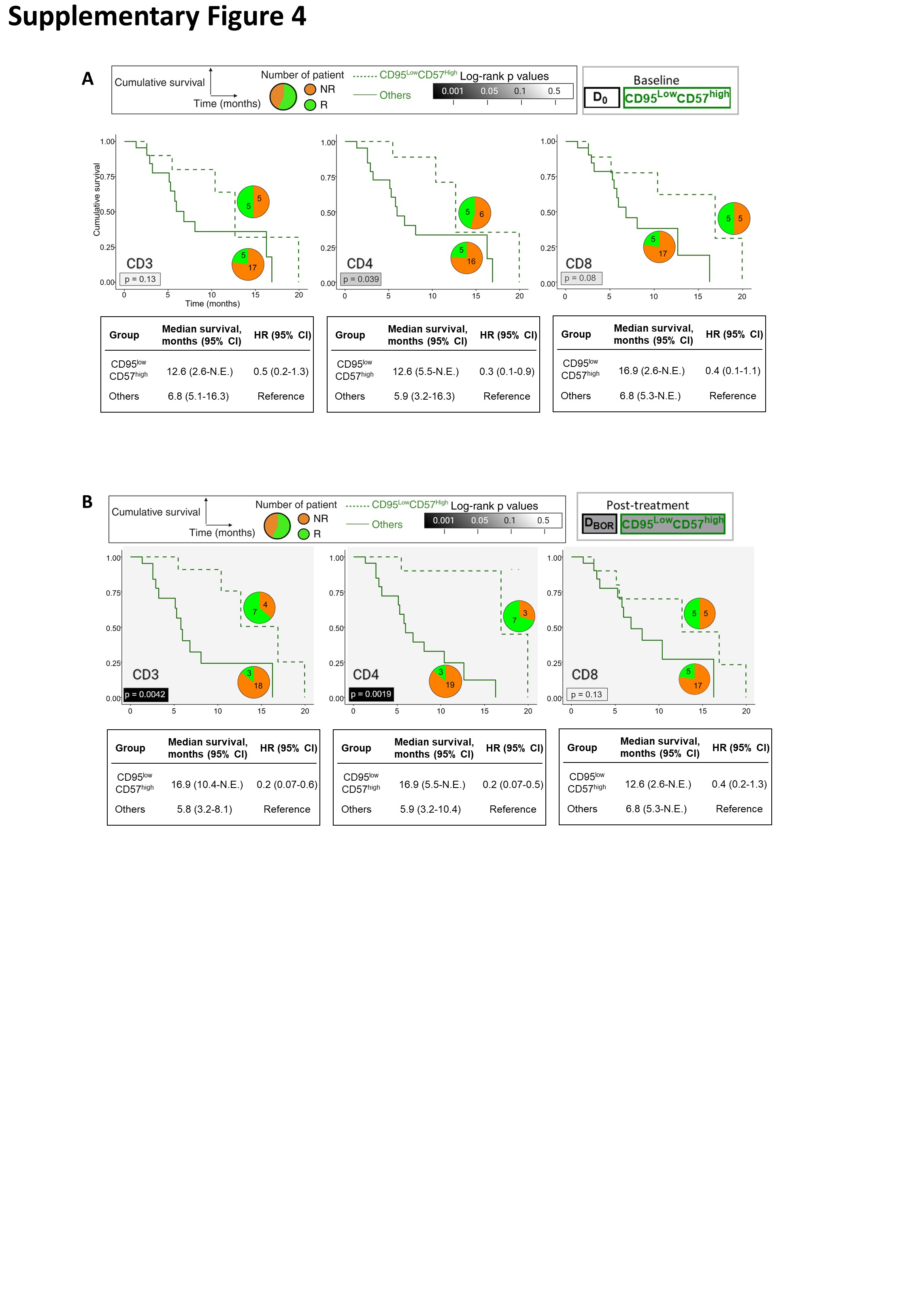

Supplement: Supplementary file 4 — Supporting Information [file CTM2-15-e70535-s002.jpg]

# Supplementary Figure 1

A

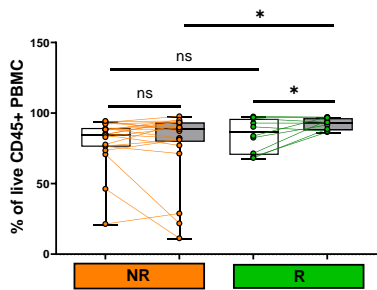

B

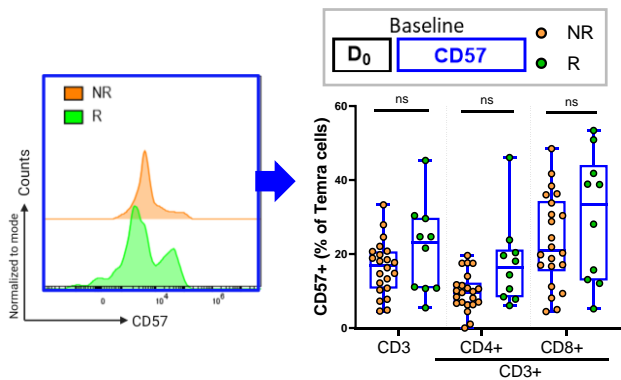

C

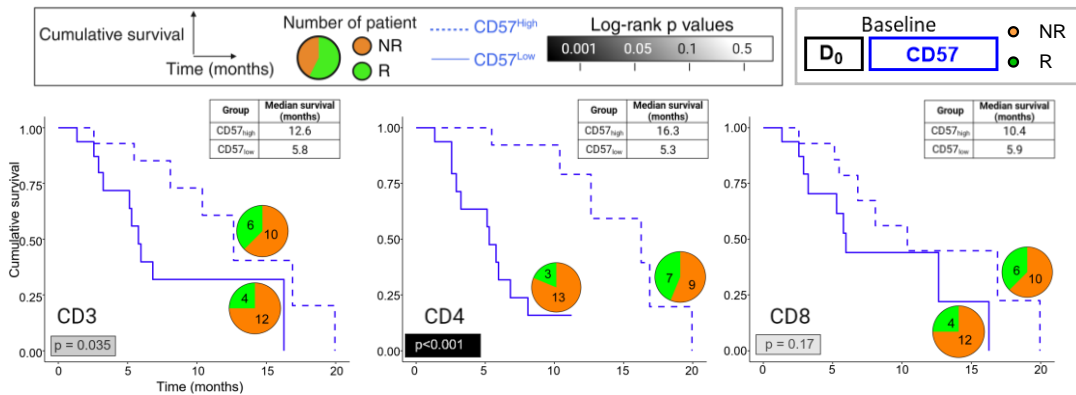

# Supplementary Figure 2

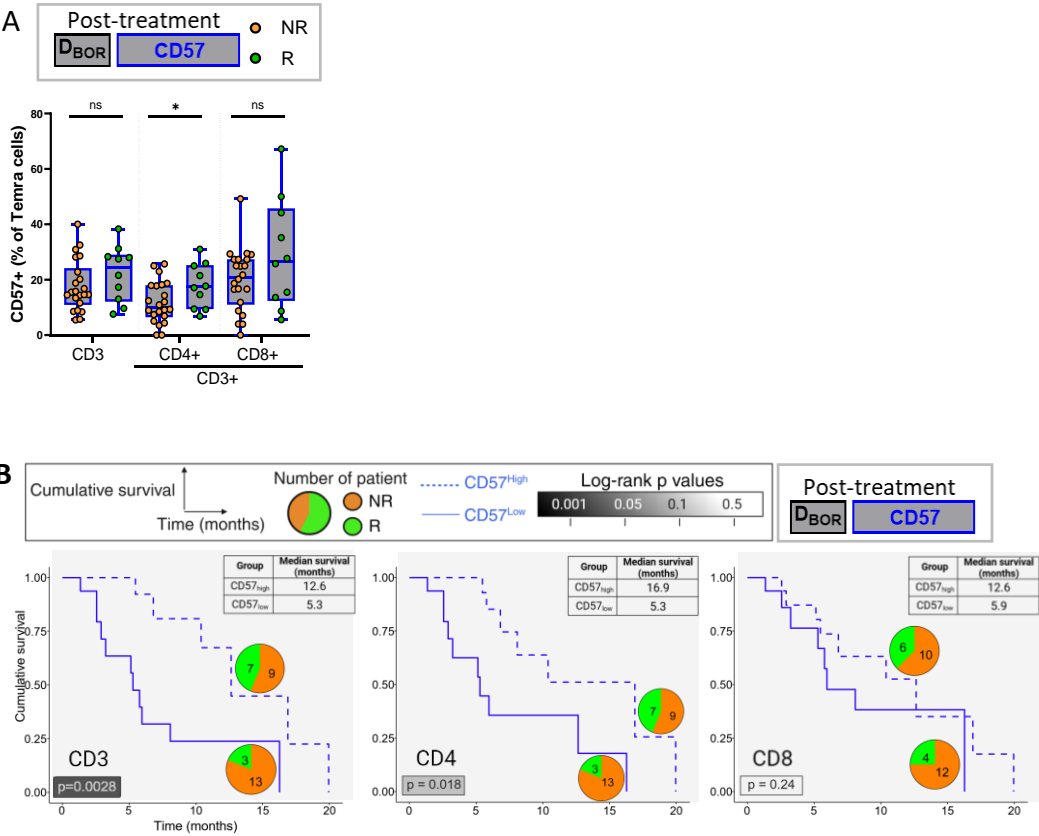

# Supplementary Figure 3

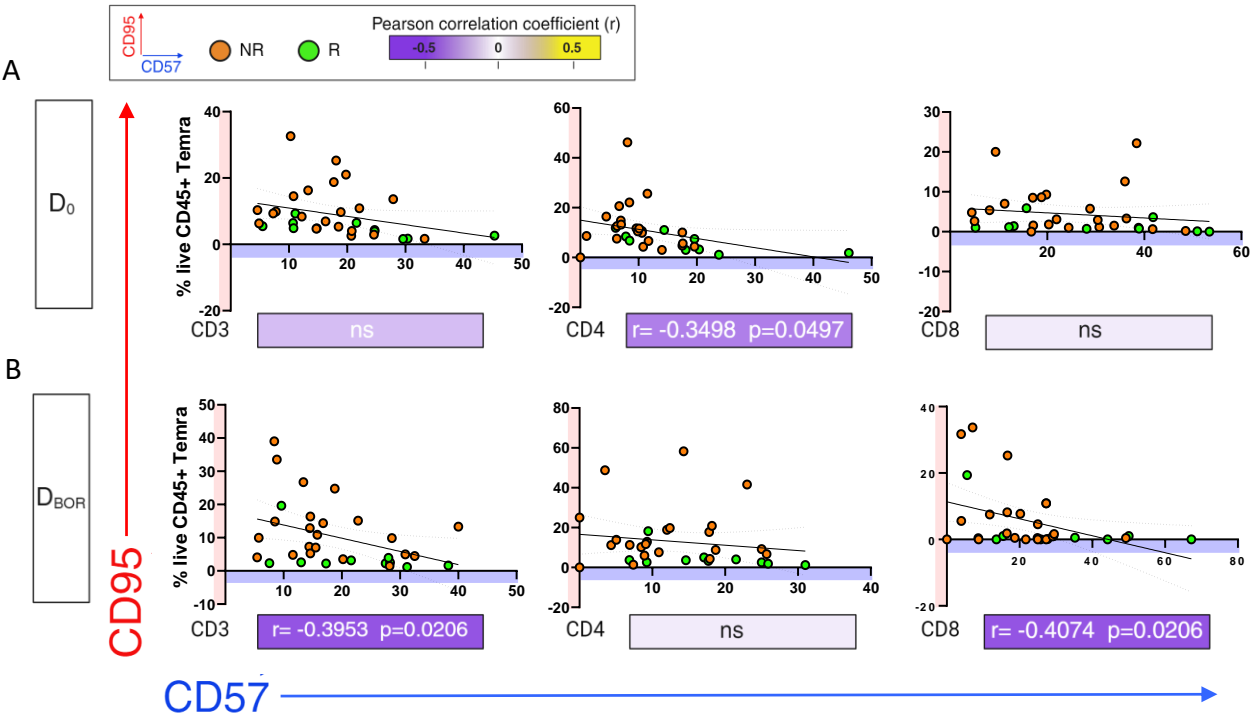

Supplementary Figure 4

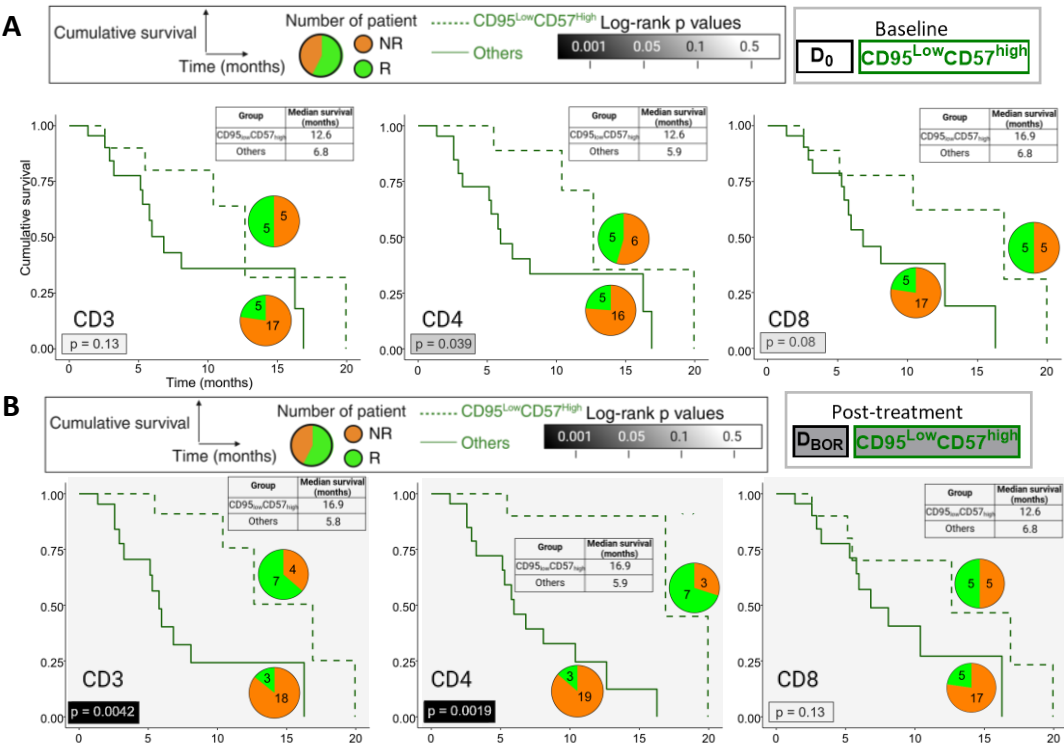

Supplement: Supplementary file 5 — Supporting Information [file CTM2-15-e70535-s005.pdf]
