# Supplementary material for: Pre‐existing CD95‐based Temra immunity in patients with recurrent/metastatic nasopharyngeal carcinoma predicts response and hyperprogression to dual PD‐L1 and TGFβ inhibition
Source: Clin Transl Med. 2025 Nov 20;15(11):e70535. doi: 10.1002/ctm2.70535 (PMC12632158; doi:10.1002/ctm2.70535)
Supplement: Supplementary file 6 — Supporting Information [file CTM2-15-e70535-s007.docx]

| **Clinical outcomes** | **No. of patients (%)** |
| --- | --- |
| Complete response (CR) | 2 (6) |
| Partial response (PR) | 6 (19) |
| Stable disease (SD) | 2 (6) |
| Progressive disease (PD) | 22 (69) |
| Death | 0 (0) |

**Supplementary Table 1.** Treatment response data to bintrafusp alfa

| **Clinical characteristics** | **N = 32 (%)** |
| --- | --- |
| Age, median (range), years | 54.5 (18-70) |
| Sex |  |
| Male | 30 (93.75) |
| Female | 2 (6.25) |
| Smoking status |  |
| Non-smoker | 16 (50) |
| Current/former smoker | 16 (50) |
| Hyperprogression |  |
| No | 18 (56.25) |
| Yes | 14 (43.75) |
| T stage at diagnosis |  |
| T1 | 6 (18.8) |
| T2 | 4 (12.5) |
| T3 | 13 (40.6) |
| T4 | 9 (28.1) |
| N stage at diagnosis |  |
| N0 | 1 (3.1) |
| N1 | 5 (15.6) |
| N2 | 16 (50) |
| N3 | 10 (31.3) |
| M stage at diagnosis |  |
| M0 | 16 (50) |
| M1 | 16 (50) |

**Supplementary Table 2:** Patients clinical characteristics before treatment. n=Total number of patients.

| **Specificity** | **Fluorochrome** | **Reagent Description** | **Status** | **Clone** | **Catalog Number** |
| --- | --- | --- | --- | --- | --- |
| CD45 | BV786 | BD Horizon™ BV786 Mouse Anti-Human CD45 | RUO | HI30 | 563716 |
| CD3 | Alexa Fluor 700 | BD Pharmingen™ Alexa Fluor® 700 Mouse Anti-Human CD3 0.1 mg | RUO | UCHT1 | 557943 |
| CD4 | BV510 | BD Horizon™ BV510 Mouse Anti-Human CD4 100 Tests | RUO | SK3 (also known as Leu3a) | 562970 |
| CD197 (CCR7) | BV421 | [BD Horizon™ BV421 Mouse Anti-Human CD197 (CCR7)](http://www.bdbiosciences.com/us/applications/research/t-cell-immunology/th-2-cells/surface-markers/human/bv421-mouse-anti-human-cd197-ccr7-150503/p/562555) | RUO | 150503 | 562555 |
| CD57 | PE-CF594 | BD Horizon™ PE-CF594 Mouse Anti-Human CD57 100 Tests | RUO | NK-1 | 562488 |
| CD45RA | PE-Cy5 | BD Pharmingen™ PE-Cy™5 Mouse Anti-Human CD45RA 25 Tests | RUO | HI100 | 561885 |
| CD8 | APC-H7 | BD Pharmingen™ APC-H7 Mouse anti-Human CD8 | RUO | SK1 | 560273 |
| CD95 (Fas) | PE-Cy7 | BD Pharmingen™ PE-Cy™7 Mouse Anti-Human CD95 50 Tests | RUO | DX2 | 561633 |
| 7AAD |  | BD Pharmingen™ 7-AAD | RUO |  | 559925 |

**Supplementary table 3.** List of antibodies used for flow cytometry analysis.

| **Phenotype** | | **Grouping** | | **Median** | **stdev** | **First quantile** | **Third.quantile** | **<= median** | **>median** | **total** |
| --- | --- | --- | --- | --- | --- | --- | --- | --- | --- | --- |
| CD3 | Naive | NR | Baseline | 3.475 | 5.168713 | 1.32 | 7.9125 | 11 | 11 | 22 |
|  |  |  | DBOR | 4.535 | 3.670838 | 1.57 | 7.415 | 11 | 11 | 22 |
|  |  | R | Baseline | 6.94 | 8.342885 | 2.8125 | 9.9 | 5 | 5 | 10 |
|  |  |  | DBOR | 4.935 | 4.666139 | 1.635 | 6.9375 | 5 | 5 | 10 |
|  | Tcm | NR | Baseline | 2.03 | 2.989322 | 1.3325 | 2.8775 | 11 | 11 | 22 |
|  |  |  | DBOR | 2.16 | 4.824654 | 1.205 | 2.605 | 11 | 11 | 22 |
|  |  | R | Baseline | 3.32 | 2.569356 | 1.0975 | 4.66 | 5 | 5 | 10 |
|  |  |  | DBOR | 2.7 | 1.89955 | 2.0975 | 3.8425 | 5 | 5 | 10 |
|  | Tem | NR | Baseline | 22.15 | 15.34505 | 17.275 | 36.175 | 11 | 11 | 22 |
|  |  |  | DBOR | 25.25 | 12.5961 | 18.55 | 36 | 11 | 11 | 22 |
|  |  | R | Baseline | 18.65 | 9.48301 | 14.5 | 24.825 | 5 | 5 | 10 |
|  |  |  | DBOR | 13.05 | 9.709039 | 12.6 | 28.675 | 5 | 5 | 10 |
|  | Temra | NR | Baseline | 67.1 | 14.15548 | 59.475 | 72.175 | 11 | 11 | 22 |
|  |  |  | DBOR | 66.55 | 13.38126 | 56.15 | 72.975 | 11 | 11 | 22 |
|  |  | R | Baseline | 67.3 | 11.72699 | 63.25 | 71.225 | 5 | 5 | 10 |
|  |  |  | DBOR | 76.5 | 13.40872 | 65.7 | 78.15 | 6 | 4 | 10 |
| CD4 | Naive | NR | Baseline | 6.025 | 9.465785 | 0.9625 | 14.325 | 11 | 11 | 22 |
|  |  |  | DBOR | 11.6 | 8.168933 | 5.055 | 17.3 | 11 | 11 | 22 |
|  |  | R | Baseline | 9.355 | 13.74783 | 3.89 | 19.525 | 5 | 5 | 10 |
|  |  |  | DBOR | 7.595 | 8.491385 | 1.4675 | 10.8 | 5 | 5 | 10 |
|  | Tcm | NR | Baseline | 1.245 | 3.092248 | 0.0375 | 4.0175 | 11 | 11 | 22 |
|  |  |  | DBOR | 1.145 | 2.134977 | 0.73 | 3.205 | 11 | 11 | 22 |
|  |  | R | Baseline | 3.8 | 3.574389 | 1.515 | 7.15 | 5 | 5 | 10 |
|  |  |  | DBOR | 4.015 | 2.369712 | 1.8975 | 4.9075 | 5 | 5 | 10 |
|  | Tem | NR | Baseline | 11.8 | 14.10975 | 5.245 | 23.2 | 11 | 11 | 22 |
|  |  |  | DBOR | 11.65 | 12.46409 | 5.2875 | 20.925 | 11 | 11 | 22 |
|  |  | R | Baseline | 10.335 | 7.94525 | 5.6425 | 14.75 | 5 | 5 | 10 |
|  |  |  | DBOR | 8.34 | 6.988874 | 6.7625 | 17.105 | 5 | 5 | 10 |
|  | Temra | NR | Baseline | 70.4 | 20.81122 | 63.125 | 79.725 | 11 | 11 | 22 |
|  |  |  | DBOR | 71.75 | 11.57405 | 65.725 | 78.525 | 11 | 11 | 22 |
|  |  | R | Baseline | 72.7 | 14.44805 | 70.025 | 76.55 | 5 | 5 | 10 |
|  |  |  | DBOR | 79.25 | 15.30029 | 73.475 | 85.375 | 5 | 5 | 10 |
| CD8 | Naive | NR | Baseline | 1.32 | 3.755469 | 0.3075 | 2.92 | 11 | 11 | 22 |
|  |  |  | DBOR | 1.375 | 3.174215 | 0.6525 | 2.42 | 11 | 11 | 22 |
|  |  | R | Baseline | 2.305 | 8.690325 | 0.645 | 3.63 | 5 | 5 | 10 |
|  |  |  | DBOR | 0.74 | 5.620686 | 0 | 2.465 | 5 | 5 | 10 |
|  | Tcm | NR | Baseline | 0.845 | 1.15624 | 0.195 | 1.585 | 11 | 11 | 22 |
|  |  |  | DBOR | 0.625 | 1.102018 | 0.155 | 1.4 | 11 | 11 | 22 |
|  |  | R | Baseline | 2.025 | 3.630917 | 0.3675 | 2.6725 | 5 | 5 | 10 |
|  |  |  | DBOR | 0.74 | 3.821434 | 0.145 | 1.0825 | 5 | 5 | 10 |
|  | Tem | NR | Baseline | 27.75 | 10.63524 | 22.8 | 34.725 | 11 | 11 | 22 |
|  |  |  | DBOR | 28.2 | 11.91206 | 21.475 | 39.7 | 11 | 11 | 22 |
|  |  | R | Baseline | 24.45 | 15.34753 | 22.125 | 36.775 | 5 | 5 | 10 |
|  |  |  | DBOR | 22.3 | 9.940914 | 21.625 | 32.925 | 5 | 5 | 10 |
|  | Temra | NR | Baseline | 68.95 | 10.20021 | 57.8 | 72.3 | 11 | 11 | 22 |
|  |  |  | DBOR | 67.35 | 11.60644 | 57.2 | 72.425 | 11 | 11 | 22 |
|  |  | R | Baseline | 63.9 | 16.78964 | 48.475 | 71.1 | 5 | 5 | 10 |
|  |  |  | DBOR | 74.95 | 14.31074 | 60.025 | 78.125 | 5 | 5 | 10 |

**Supplementary table 4.** T-cell memory population in patients group of responders (R) and non-responders (NR) on screening (baseline) and on-treatment (at Day of best overall response, DBOR). Tn, naïve; Tcm, T-cell central memory; Tem, T-cell effector memory; Temra, T-cell effector memory re-expressing CD45RA.

| **CD95 (D0)** | | | | | | | | | |
| --- | --- | --- | --- | --- | --- | --- | --- | --- | --- |
|  | **Phenotype** | **Grouping** | **Median** | **stdev** | **First quantile** | **Third quantile** | **<= Median** | **>Median** | **Total** |
| **CD3** |  | NR | 8.44 | 7.854399 | 6.3875 | 12.125 | 11 | 11 | 22 |
|  |  | R | 3.18 | 2.614384 | 1.4575 | 4.9125 | 5 | 5 | 10 |
|  | Naive | NR | 22.25 | 17.90251 | 16.8 | 36.825 | 11 | 11 | 22 |
|  |  | R | 21.75 | 10.7971 | 14.3 | 24.375 | 5 | 5 | 10 |
|  | Tcm | NR | 15.05 | 17.65749 | 1.9875 | 28.875 | 11 | 11 | 22 |
|  |  | R | 11.39 | 17.18351 | 7.595 | 18.75 | 5 | 5 | 10 |
|  | Tem | NR | 2.46 | 8.035906 | 0.955 | 10.02 | 11 | 11 | 22 |
|  |  | R | 3.03 | 2.739842 | 1.135 | 4.4875 | 5 | 5 | 10 |
|  | Temra | NR | 9.53 | 7.904007 | 4.93 | 14.275 | 11 | 11 | 22 |
|  |  | R | 4.605 | 2.376122 | 2.9475 | 6.18 | 5 | 5 | 10 |
| **CD4** |  | NR | 11.5 | 10.37315 | 4.765 | 16.425 | 11 | 11 | 22 |
|  |  | R | 7.64 | 8.119657 | 6.015 | 17.275 | 5 | 5 | 10 |
|  | Naive | NR | 13.25 | 29.27359 | 0 | 41.175 | 11 | 11 | 22 |
|  |  | R | 12.45 | 14.60251 | 1.745 | 22.65 | 5 | 5 | 10 |
|  | Tcm | NR | 0 | 19.398 | 0 | 8.025 | 16 | 6 | 22 |
|  |  | R | 0 | 5.641501 | 0 | 5.32 | 6 | 4 | 10 |
|  | Tem | NR | 0 | 3.477192 | 0 | 2.605 | 14 | 8 | 22 |
|  |  | R | 1.625 | 3.675164 | 0 | 2.6375 | 5 | 5 | 10 |
|  | Temra | NR | 10.25 | 9.91133 | 5.8975 | 14.4 | 11 | 11 | 22 |
|  |  | R | 7.105 | 4.009259 | 3.0875 | 10.0525 | 5 | 5 | 10 |
| **CD8** |  | NR | 3.505 | 6.299385 | 1.7025 | 8.95 | 11 | 11 | 22 |
|  |  | R | 1.295 | 1.048345 | 0.6125 | 1.9225 | 5 | 5 | 10 |
|  | Naive | NR | 0 | 14.5982 | 0 | 6.525 | 16 | 6 | 22 |
|  |  | R | 0 | 20.8922 | 0 | 2.4225 | 7 | 3 | 10 |
|  | Tcm | NR | 0 | 25.40346 | 0 | 0 | 18 | 4 | 22 |
|  |  | R | 0 | 10.48519 | 0 | 0.885 | 7 | 3 | 10 |
|  | Tem | NR | 2.165 | 7.074974 | 0 | 9.345 | 11 | 11 | 22 |
|  |  | R | 1.035 | 1.330729 | 0 | 2.325 | 5 | 5 | 10 |
|  | Temra | NR | 3.21 | 6.041693 | 1.545 | 8.1375 | 11 | 11 | 22 |
|  |  | R | 0.985 | 1.817821 | 0.7375 | 1.3175 | 5 | 5 | 10 |

**Supplementary table 5.** Frequencies of CD95-expressingTemra cells in patients group of responders (R) and non-responders (NR) before-treatment. Tn, naïve; Tcm, T-cell central memory; Tem, T-cell effector memory; Temra, T-cell effector memory re-expressing CD45RA.

| **CD95 (DBOR)** | | | | | | | | | |
| --- | --- | --- | --- | --- | --- | --- | --- | --- | --- |
|  | **phenotype** | **grouping** | **median** | **stdev** | **first.quantile** | **third.quantile** | **<= median** | **> median** | **total** |
| CD3 |  | NR | 2.625 | 5.825096 | 1.21 | 4.0175 | 11 | 11 | 22 |
|  |  | R | 2.51 | 2.32618 | 2.035 | 2.7625 | 5 | 5 | 10 |
|  | Naive | NR | 22.45 | 18.44583 | 14.975 | 37.4 | 11 | 11 | 22 |
|  |  | R | 23.2 | 29.05012 | 10.815 | 30.25 | 5 | 5 | 10 |
|  | Tcm | NR | 9.11 | 17.94313 | 7.32 | 20.5 | 11 | 11 | 22 |
|  |  | R | 0 | 8.091648 | 0 | 3.9 | 6 | 4 | 10 |
|  | Tem | NR | 4.19 | 12.63662 | 1.35 | 14.3 | 11 | 11 | 22 |
|  |  | R | 2.06 | 4.593746 | 1.48 | 3.0925 | 5 | 5 | 10 |
|  | Temra | NR | 10.44 | 10.00645 | 5.065 | 15.05 | 11 | 11 | 22 |
|  |  | R | 2.42 | 5.484681 | 2.23 | 3.015 | 5 | 5 | 10 |
| CD4 |  | NR | 11.95 | 13.30683 | 7.305 | 19.725 | 11 | 11 | 22 |
|  |  | R | 3.59 | 5.780123 | 2.595 | 6.1675 | 5 | 5 | 10 |
|  | Naive | NR | 19.85 | 27.6042 | 10.875 | 45.2 | 11 | 11 | 22 |
|  |  | R | 9.29 | 17.56074 | 0 | 21.275 | 5 | 5 | 10 |
|  | Tcm | NR | 0 | 21.44343 | 0 | 5.3575 | 15 | 7 | 22 |
|  |  | R | 0 | 7.127477 | 0 | 1.29 | 7 | 3 | 10 |
|  | Tem | NR | 0 | 3.998043 | 0 | 1.1775 | 13 | 9 | 22 |
|  |  | R | 1.41 | 5.439948 | 0 | 6.2525 | 5 | 5 | 10 |
|  | Temra | NR | 11.5 | 14.9875 | 7.93 | 19.575 | 11 | 11 | 22 |
|  |  | R | 3.48 | 4.896251 | 2.5875 | 3.9275 | 5 | 5 | 10 |
| CD8 |  | NR | 2.67 | 11.39603 | 0.7075 | 9.785 | 11 | 11 | 22 |
|  |  | R | 0.38 | 5.654018 | 0 | 0.73 | 5 | 5 | 10 |
|  | Naive | NR | 0 | 14.42573 | 0 | 19.9 | 14 | 8 | 22 |
|  |  | R | 0 | 16.78357 | 0 | 1.56 | 7 | 3 | 10 |
|  | Tcm | NR | 0 | 13.77071 | 0 | 7.5 | 16 | 6 | 22 |
|  |  | R | 0 | 0 | 0 | 0 | 10 | 0 | 10 |
|  | Tem | NR | 2.805 | 13.5583 | 0 | 9.985 | 11 | 11 | 22 |
|  |  | R | 0 | 4.871667 | 0 | 0 | 8 | 2 | 10 |
|  | Temra | NR | 1.735 | 10.23644 | 0.4325 | 8.0475 | 11 | 11 | 22 |
|  |  | R | 0.265 | 6.039311 | 0 | 0.9 | 5 | 5 | 10 |

**Supplementary table 6**. Frequencies of CD95-expressingTemra cells in patients group of responders (R) and non-responders (NR) on treatment (at Day of best overall response, DBOR). Tn, naïve; Tcm, T-cell central memory; Tem, T-cell effector memory; Temra, T-cell effector memory re-expressing CD45RA.

| **CD57 (DBOR)** | | | | | | | | | |
| --- | --- | --- | --- | --- | --- | --- | --- | --- | --- |
|  | **phenotype** | **grouping** | **median** | **stdev** | **first.quantile** | **third.quantile** | **<= median** | **> median** | **total** |
| CD3 |  | NR | 16.25 | 10.72616723 | 11.1 | 28.7 | 11 | 11 | 22 |
|  |  | R | 25.35 | 12.18596296 | 14.775 | 33.85 | 5 | 5 | 10 |
|  | Naive | NR | 26.6 | 12.23560998 | 19.075 | 31 | 11 | 11 | 22 |
|  |  | R | 30.65 | 33.32342519 | 18.35 | 59.575 | 5 | 5 | 10 |
|  | Tcm | NR | 31.2 | 21.10211669 | 19.9 | 47.475 | 11 | 11 | 22 |
|  |  | R | 51.7 | 33.5858105 | 16.65 | 75.425 | 5 | 5 | 10 |
|  | Tem | NR | 20.05 | 12.72570774 | 11.75 | 30.875 | 11 | 11 | 22 |
|  |  | R | 28.95 | 16.88999339 | 16.7 | 45.7 | 5 | 5 | 10 |
|  | Temra | NR | 15.05 | 9.259045899 | 12.05 | 22.15 | 11 | 11 | 22 |
|  |  | R | 24.55 | 10.10990982 | 14.075 | 28.225 | 5 | 5 | 10 |
| CD4 |  | NR | 11.55 | 7.380738123 | 8.5875 | 17.95 | 11 | 11 | 22 |
|  |  | R | 17.85 | 10.34969865 | 11.4125 | 29.4 | 5 | 5 | 10 |
|  | Naive | NR | 19.5 | 13.99547333 | 1.5625 | 25.025 | 11 | 11 | 22 |
|  |  | R | 23.7 | 33.63133783 | 11.11 | 37.225 | 5 | 5 | 10 |
|  | Tcm | NR | 4.84 | 15.11417617 | 0 | 15.75 | 11 | 11 | 22 |
|  |  | R | 33.6 | 38.59703644 | 8.5875 | 72.5 | 5 | 5 | 10 |
|  | Tem | NR | 10.31 | 21.1989534 | 4.55 | 15.7 | 11 | 11 | 22 |
|  |  | R | 18.9 | 20.9740602 | 12.9 | 50.025 | 5 | 5 | 10 |
|  | Temra | NR | 10.085 | 7.519429264 | 7.02 | 17.875 | 11 | 11 | 22 |
|  |  | R | 17.4 | 8.02437045 | 10.7225 | 24.125 | 5 | 5 | 10 |
| CD8 |  | NR | 22.15 | 11.87050315 | 16 | 29.1 | 11 | 11 | 22 |
|  |  | R | 25.15 | 16.92993614 | 14.05 | 41 | 5 | 5 | 10 |
|  | Naive | NR | 13.645 | 17.87494087 | 0 | 31.9 | 11 | 11 | 22 |
|  |  | R | 0 | 8.449077859 | 0 | 4.6875 | 7 | 3 | 10 |
|  | Tcm | NR | 0 | 31.53339582 | 0 | 27 | 13 | 9 | 22 |
|  |  | R | 4.165 | 32.8837264 | 0 | 27.2475 | 5 | 5 | 10 |
|  | Tem | NR | 22.5 | 21.23929504 | 13.35 | 33 | 11 | 11 | 22 |
|  |  | R | 20.65 | 12.78932823 | 18.175 | 37.325 | 5 | 5 | 10 |
|  | Temra | NR | 20.95 | 11.25526143 | 12.975 | 27.3 | 11 | 11 | 22 |
|  |  | R | 26.75 | 19.89822239 | 14.075 | 41.95 | 5 | 5 | 10 |

**Supplementary table 7.** Frequencies of CD57-expressingTemra cells in patients group of responders (R) and non-responders (NR) on treatment (at Day of best overall response, DBOR). Tn, naïve; Tcm, T-cell central memory; Tem, T-cell effector memory; Temra, T-cell effector memory re-expressing CD45RA.

**Supplemetnary Figure legends**

**Supplementary Figure 1**

(**A**) Boxplots illustrate the frequencies of CD45+ lymphocytes for total responders (n=10) and non-responder patients (n=22) compared timepoints between D0 and DBOR Example staining of CD57 on gated live CD45+ lymphocytes in responding vs. non‐responding NPC patients. (**B**) Frequencies of CD57 on gated Temra cells in each population for total responder and non-responder patients. (**C**) Kaplan–Meier plot showing overall survival of patients with CD3/4/8+ Temra cell %CD57 expression above and below the median. ns, non-significant. Data is displayed with treatment response groups and timepoints colour-coded; responders, green; non-responder, orange; D0, white. In the boxplots, the centerline indicates the median, with upper and lower quartile ranges. Statistically significant differences between BOR groups were determined using unpaired T test. Log-rank analysis was employed to evaluate the prognosis and p value are color coded as indicated.

**Supplementary Figure 2**

(**A**) On-treatment frequencies of CD57 gated Temra cells in each population for total responder (n = 10) and non-responder patients (n = 22). The mean percentages of CD57 expression for CD3+ cells were 15.0% for non-responders and 24.6% for responders (p=0.2243), for CD3+CD4+ cells were 10.1% for non-responders and 17.1% for responders (p=0.0496), and for CD3+CD8+ cells were 21.0% for non-responders and 26.8% for responders (p=0.0992). (**B**) The overall survival comparison with CD3/4/8+ Temra cell between the high- and low- subgroups (above and below the median) with regard to %CD57. High D_BOR_ vs. low CD57 Temra cells median OS (months): 12.6 vs. 5.3 for total CD3+ cells (p=0.0028), 16.9 vs. 5.3 in CD3+CD4+ cells (p=0.018), and 12.6 vs. 5.9 months in CD3+CD8+ cells (p=0.24). *p<0.05, ns, non-significant. Data is displayed with treatment response groups and timepoints colour-coded; responders, green; non-responder, orange; DBOR, shaded; CD57, blue. In the boxplots, the centerline indicates the median, with upper and lower quartile ranges. Statistically significant differences between BOR groups were determined using unpaired T test. Log-rank analysis was employed to evaluate the prognosis and p value are color coded as indicated.

**Supplementary Figure 3**

Correlation showing Spearman r values for correlations between levels of %CD57 and %CD95 expressing Temra cells. Correlation coefficient values are color coded as indicated.

**Supplementary Figure 4**

The overall survival comparison with CD3/4/8+ Temra cell between the high- and low- subgroups (above and below the median) with regard to %CD95low CD57high expression. D_0_ median OS (months): 12.6 vs.5.9, p=0.039 and D_BOR_: 16.9 vs 5.9, p=0.0019. Data is displayed with treatment response groups and timepoints colour-coded; responders, green; non-responder, orange. Log-rank analysis was employed to evaluate the prognosis and p value are color coded as indicated.
