# Supplementary material for: Pre‐existing CD95‐based Temra immunity in patients with recurrent/metastatic nasopharyngeal carcinoma predicts response and hyperprogression to dual PD‐L1 and TGFβ inhibition
Source: Clin Transl Med. 2025 Nov 20;15(11):e70535. doi: 10.1002/ctm2.70535 (PMC12632158; doi:10.1002/ctm2.70535)
Supplement: Supplementary file 7 — Supporting Information [file CTM2-15-e70535-s004.docx]

**Methods**

**Study Population and Assessments of Clinical Outcomes**

This is a companion correlative study looking for biomarkers in peripheral immune cells predictive of response to bintrafusp alfa. Study design of the clinical trial was reported before. Briefly, NCT04396886 was a single arm, prospective single-centre phase II study evaluating the antitumoral activity of bintrafusp alfa in patients with heavily pretreated R/M NPC. Patients presenting with isolated local recurrence or disease progression were not included. The trial was sponsored by Merck Healthcare, which provided the study drug. Study protocols, including amendments, received approval from the relevant institutional review boards or ethics committees. Written informed consent was obtained from each participant prior to enrollment. Patients with histologically confirmed NPC, who had at least one prior line of platinum-based chemotherapy for recurrent disease, were subsequently treated with 1200 mg bintrafusp alfa every two weeks until disease progression, unacceptable toxicities, or upon withdrawal of consent. All patients had measurable disease at baseline. Peripheral blood was collected from patients on the day of the first treatment and every two weeks before each dose of bintrafusp alfa. These samples correspond to the ‘baseline’ and ‘on treatment’ time points. 32 patients were chosen for this study based on the availability of PBMCs from paired peripheral blood collected at baseline and at best response assessment for analysis and correlation with clinical response. Plasma TGFβ was measured at week 8.

**Response Assessment**

Tumor response assessment was performed using Response Evaluation Criteria in Solid Tumors (RECIST) v. 1.1 criteria. An independent radiological assessment was performed every 8 weeks for the first 12 months and then every 12 weeks thereafter until confirmation of disease progression. The best overall response (BOR) achieved on treatment with bintrafusp alfa was recorded for RECIST according to the following categories: complete response (CR), partial response (PR), stable disease (SD), progressive disease (PD). For comparison purposes, we grouped patients as responders and non-responders. Responders included patients with complete response (CR), partial response (PR) or stable disease (SD) for at least 6 months on treatment. Non-responders were patients who had PD or SD for less than 6 months.

**Hyperprogression assessment**

Post-hoc analyses of hyperprogression (HP) incidence were conducted using two approaches. First, the RECIST 1.1 criteria, which indicated progressive disease within the initial 8 weeks after the start of treatment, characterized by a minimum increase of 10 mm in measurable lesions, along with either: (1) a ≥40% rise in the total sum of target lesions compared to baseline, or (2) a ≥20% rise in the total sum of target lesions compared to baseline with new lesions appearing in at least two distinct organs. Second, HP was assessed based on doubling of the tumor growth rate (TGR) following treatment initiation. Tumor growth (TG) is calculated using: TG = 3 Log (Dt/D0)/t, where (D) represents tumor size defined by the sum of the longest diameters of target lesions per RECIST 1.1. TGR was then expressed as a monthly percentage increase in tumor volume using: TGR = 100 (exp (TG) − 1), where exp (TG) denotes the exponential of TG.

**Blood Collection and Isolation of Peripheral Blood Mononuclear Cells**

In order to evaluate the T-cell landscape of circulating immune cells before and during therapy, 20ml blood was collected at baseline and at subsequent on treatment time points in K2EDTA (dikalium salt of ethylenediaminetetraacetic acid) tubes. Peripheral blood mononuclear cells (PBMC) were isolated by Ficoll–Paque (GE Healthcare) density gradient centrifugation. Single-cell suspensions were obtained, and after lysis of red blood cells using ammonium-chloride-potassium buffer, PBMCs were cryopreserved in 10% dimethylsulfoxide (Sigma-Aldrich), 90% fetal calf serum (Gibco, ThermoFisher) for further analysis.

**Plasma TGFβ1 detection**

After blood collection, samples were immediately placed on ice and centrifuged at 3000g for 30 minutes at 4°C within 6 hours. Plasma supernatants were collected and stored at -80°C before use. TGFβ concentrations were determined using a specific enzyme-linked immunosorbent assay (Human TGFβ DuoSet kit, R&D Systems Inc).

**Biomarker study**

Quantification of plasma Epstein-Barr virus (EBV) DNA levels was performed using real-time quantitative polymerase chain reaction (qPCR) targeting EBV-specific gene sequences. Blood samples were collected before treatment and subsequently at two-weeks intervals until disease progression.

**Flow cytometry**

Flow cytometry staining was carried out using cryopreserved PBMC samples. After thawing, the cells were incubated at 37°C overnight in RPMI1640 medium supplemented with 10% FCS and Penicillin/Streptomycin. Cells were then stained with a mixture of antibodies for 30 minutes at 4°C including 7-AAD in order to identify dead cells. Online supplementary table 1 lists the antibodies used for the stainings. Antibodies were purchased from BD Bioscience. Distinct T cell maturation subsets were characterized based on the expression of surface markers CD45RA and CCR7. These included CD45RA+CCR7+ naive T cells (Tn), CD45RA−CCR7+ central memory T cells (Tcm), CD45RA−CCR7− effector memory T cells (Tem) and CD45RA+CCR7− effector memory re-expressing RA T cells (Temra). These populations were analyzed within the CD3+(total), CD3+CD4+ and CD3+CD8+ compartments. Samples acquisition was carried out using with NovoCyte Quanteon flow cytometer, and data processed with FlowJo v10.1 software (BD Biosciences). The gating strategy followed the sequence: SSC-A vs FSC-A to gate for lymphocytes 🡪 FSC-H vs FSC-W to gate for single cells--> CD45/Live_Dead to select viable total immune cells 🡪 CD3 to gated for T cells 🡪 CD4 or CD8 within CD3+ to distinguish CD4+ or CD8+ subsets. Further gating was refined using fluorescenceminus-one (FMO) controls. Gating strategy is shown in Figure S1A.

**sPLS-DA analysis**

Sparse partial least squares discriminant analysis (sPLS-DA) was carried out with the mixOmics R package (v6.32.0) to identify features that best discriminate between the responder and non-responder groups. The number of variables per component was optimized using 10 times repeated 5-fold cross-validation. Model performance was evaluated using the balanced error rate (BER) and AUC. The final model was used to extract feature loadings and visualize sample separation, and all results, including component score plots, 95 % confidence ellipses, density plot and loading barplots, were visualized using ggplot2 (v3.5.2)

**Biomarker Stratification and Quadrant Analysis**

To investigate the combined impact of plasma TGFβ levels and CD95⁺ Temra cell frequencies on clinical outcomes, we performed a quadrant-based stratification analysis. Cut-off values for each biomarker were determined using cohort-derived 95% confidence intervals (CI). Specifically: TGFβ low was defined as plasma levels below he lower bound of the 95% CI, CD95⁺ Temra high was defined as cell frequencies above the upper bound of the 95% CI. These thresholds were used to stratify patients into four quadrants based on their combined biomarker profiles, enabling identification of a subgroup (TGFβlow/ CD95⁺ Temrahigh) associated with hyperprogression (HP).

**Outcomes and statistical analysis**

Descriptive statistics were used to examine the distributions of immune biomarker and patients' characteristics, reported as mean, median and range (min-max). Biomarker data were also dichotomized using median values as cut-off points to create equally-sized subgroups. All variables were expressed in terms of relative frequencies (percentages). The Kaplan-Meier method was used to assess the impact of dichotomized immune biomarkers on overall survival (OS) across T-cell subsets, with the median as the threshold to define high versus low expression groups. Survival differences were evaluated by a log-rank test. OS was defined as the time from enrollment to death from any cause. Additional post-hoc analyses on the incidence of hyper-progression (HP) were assessed, as previously described. Immune biomarker measurements at baseline (cycle 1, D_0_) and at BOR (D_BOR_) were compared according to RECIST BOR status (responding vs. non-responding). Sparse partial least squares discriminant analysis (sPLS-DA) was performed using the mixOmics R package (v6.32.0) to identify features that best discriminate between the responder and non-responder groups. Variable selection per component was optimized using 10 times repeated 5-fold cross-validation. Model performance was evaluated using the balanced error rate (BER) and AUC. The final model provided feature loadings and visualizations, including component score plots, 95 % confidence ellipses, density plot and loading barplots, were visualized using ggplot2 (v3.5.2). Odds ratio for biomarker enrichment was calculated using the Haldane–Anscombe correction and Woolf method. Association between immune biomarker measurement (D_0_ and D_BOR_) and RECIST BOR outcome were examined using Pearson correlation. Statistical significance was set at two-tailed p <0.05. Unpaired t-test compared independent groups and paired t-test was used for paired samples.
